# Responses to Sedimentation in Ramet Populations of the Clonal Plant *Carex brevicuspis*

**DOI:** 10.3389/fpls.2018.00512

**Published:** 2018-04-16

**Authors:** Bai-Han Pan, Yong-Hong Xie, Feng Li, Ye-Ai Zou, Zheng-Miao Deng

**Affiliations:** ^1^Key Laboratory of Agro-ecological Processes in Subtropical Region, Institute of Subtropical Agriculture, Chinese Academy of Sciences, Changsha, China; ^2^Dongting Lake Station for Wetland Ecosystem Research, Institute of Subtropical Agriculture, Chinese Academy of Sciences, Changsha, China; ^3^University of Chinese Academy of Sciences, Beijing, China

**Keywords:** clonal plant, biomass accumulation, ramet population, morphological plasticity, spacer length, growth form, sedimentation, Dongting Lake

## Abstract

In aquatic ecosystems, sedimentation is an important factor that affects plant growth, mainly due to sediment depth. Clonal morphological plasticity is an effective strategy in clonal plants for acclimatization to sediment burial. To date, few studies have examined growth responses to sedimentation on the clonal plants at the ramet population level. This study aimed to explore the interactive effects of population size and burial depth on growth and clonal morphology of *Carex brevicuspis*. Three population sizes (2, 8, and 32 ramets) and 3 burial depths (0 cm, 5 cm, and 10 cm) were used in this experiment. Under shallow (5 cm) and deep (10 cm) burial conditions, biomass accumulation and relative growth rate (RGR) were lower than in the no burial treatment (*P* < 0.05). RGR of the small and medium populations was especially high compared to the large populations (*P* < 0.05). Biomass allocation was higher to belowground parts than aboveground parts, except for the small populations in the 5 cm burial treatments. Both shallow burial and smaller populations led to more biomass being allocated to aboveground parts. Deep burial elongated the first order spacer more than shallow burial, and sedimentation had negative effects on the second order spacer length. The number of new ramets did not decrease in the 5 or 10 cm burial treatments compared to the unburial treatment, and larger populations usually had more ramets than smaller ones; the proportion of clumping ramets was higher than the proportion of spreading ramets, and deeper burial and smaller populations led to higher proportions of spreading ramets. These results indicated that the growth of *C. brevicuspis* was limited by sediment burial at the ramet population level. Smaller populations enable *C. brevicuspis* to adjust its escape response to burial stress, may allow this species to effectively survive and widely distribute in Dongting Lake wetland.

## Introduction

In aquatic ecosystems, such as lake wetlands and river wetlands, frequent sedimentation directly affects growth and distribution of plants (Maun, 1998; Li et al., 2009, 2016; Zheng et al., 2009). Sedimentation has multiple effects on the growth of plants, mainly due to the depth of sediment burial (Maun, 1998; Shi et al., 2004). Under deep burial conditions, growth of plants, such as biomass accumulation, relative growth rates and number of plants, are usually inhibited (Yu et al., 2004; Li and Xie, 2009; Pan et al., 2012). These limitations caused by sediment burial are mainly caused by the physical burden on the buried parts of plants (Maun, 1998; Werner and Zedler, 2002; Koning, 2004) and fluctuations in abiotic conditions, such as decreases in the light availability (Madsen et al., 2001), changes in nutrient contents, particle composition, and temperature of the soil (Xiao et al., 2010; Pan et al., 2016). However, under shallow or medium burial conditions, growth of some plants can be promoted via phenotypic plasticity, such as elongation of petioles, stem internodes, and rhizomes, and adjusting the number of ramets and the biomass allocation to aboveground parts (Maun, 1998; Deng et al., 2008; Luo and Zhao, 2015).

Clonal plants, which are dominant in wetland ecosystems, can cope with complicated burial stresses by higher levels of phenotypic plasticity compared with non-clonal plants (Dong et al., 1997; Santamaria, 2002; Li and Xie, 2009; Chen et al., 2011). In clonal plants, studies on the responses to sediment burial mostly focus on the plasticity of clonal architecture and the change in clonal growth forms, such as the length of rhizomes and the number of clumping or spreading ramets (Klimes et al., 1993; Li and Xie, 2009; Chen et al., 2011, 2017). However, clonal integration among interconnected ramets, generally aids clonal plants to endure frequently occurring sediment burial (Yu et al., 2001, 2004; Chen et al., 2010; Luo and Zhao, 2015).

Integrations of clonal plants, such as genets or fragments, refers to the interconnection of clonal network, shares the resources and information among ramets of clonal plants (Liu et al., 2016); these integrations of clonal plants can be seen as populations of ramets (Herben et al., 1994; Wang et al., 2004). At the population level, there are many studies on the responses of the clonal plants to disturbances, such as sand burial (Yu et al., 2001, 2004), defoliation (Wang et al., 2004, 2017), wind erosion (Yu et al., 2008), grazing (Liu et al., 2009), and soil nutrient heterogeneity (Zhou et al., 2012). Further, the growth responses to burial stress in clonal plants with different ramet population sizes remain unclear. In the present study, a factorial experiment was conducted to elucidate the role of ramet population size, on the growth and clonal morphology of *Carex brevicuspis*, in response to the depth of burial by sediment.

In particular, we tested the following hypotheses: (1) plant growth of *C. brevicuspis* populations will be promoted under shallow burial conditions and be limited under deep burial conditions, and larger populations will have higher biomass accumulation and relative growth rate (RGR) compared to other sizes of populations; (2) In *C. brevicuspis* populations, elongation of spacers and increase in number of ramets, biomass allocation to aboveground parts, and proportion of spreading ramets, are effective strategies to escape burial stress, and these morphological changes will be smaller in larger populations under deeper burial, compared to other sizes of population.

## Materials and Methods

### The Species

The genus *Carex*, which consists of approximately 2000 species, is important in wetland ecosystems (Bernard, 1990). The pseudoculm of *C. brevicuspis* is a series of overlapping leaf sheaths, 20–55 cm in height. This species is a perennial rhizomatous sedge and widely distributed in eastern mainland China and Taiwan (Dai et al., 2000). *C. brevicuspis* is one of the dominant species in the Dongting Lake wetland, where is the second largest freshwater lake and is connected to the Yangtze Rivers in China. This wetland is usually flooded from May to October, accrete 3–7 cm sediment annually during the flooding season (Zheng et al., 2009; Li et al., 2016). Recruitment of this plant in the Dongting Lake wetlands is mainly through vegetative ramets emerging from underground rhizomes, from which tiller clumps or tussocks of various sizes can be formed (Chen et al., 2014). Growth of *C. brevicuspis* is inhibited and more biomass is allocated to leaves under high water level (Pan et al., 2012). Two types of ramets, clumping and spreading, can be produced in response to sedimentation (Chen et al., 2011).

### Experimental Design

On June 15, 2014, mature populations of *C. brevicuspis* were transported from the sampling site (29°30′ N, 112°48′ E) of East Dongting Lake wetlands, China. They were transplanted into three outdoor concrete pools (200 cm in length and width, and 100 cm in height) at the Dongting Lake Station for Wetland Ecosystem Research, The Chinese Academy of Sciences. A two-way factorial design was used for the experiment, which combined 3 burial depths (0, 5, and 10 cm; which were referred to as control, shallow, and deep burial conditions) with three population sizes (small, medium, and large). Small, medium and large populations were consisted of 2, 8, and 32 tillers, which were constructed with clumps of 5, 10, and 20 cm. The initial biomass of plants in each population size was recorded (3.98 ± 0.29 g, 13.59 ± 1.17 g, 54.37 ± 1.26 g, respectively; mean ± SE). Each pool was divided equally into nine square blocks, using bricks (40 cm in height). Each block (63 cm in length, 40 cm in height) had 30 cm sediment placed in the bottom. Plants for each population size were then randomly transplanted in the center of each block.

On June 30, 2014, a one-time sediment addition (0, 5, or 10 cm) was made to blocks of each population size. The experiment therefore comprised, nine treatments (small, medium, and large populations, with 0, 5, and 10 cm burial), with three replicates (one in each concrete pool). Sediment used in this experiment was collected from the area with *Carex* vegetation in the east Dongting Lake (29°30′ N, 112°48′ E), containing 2.6% organic matter, 9.6 μg g^-1^ total nitrogen, and 0.13 μg g^-1^ total phosphorus). Well water (containing 30 μg L^-1^ NH_4_^+^-N, 40 μg L^-1^ NO_3_^-^-N, and 20 μg L^-1^ PO_4_^3+^-P, pH = 7.59) was added every week to maintain a 30 cm water level, relative to the pool bottom.

### Harvest and Measurements

Plants were harvested on June 30, 2015, before the plants of the large populations in the 0 cm burial treatments reached the edge of the blocks. Whole plants in each block were carefully cleaned using well water, and the number of new ramets produced by original plant were counted. The numbers of clumping or spreading ramets were counted, according to the method by Chen et al. (2011). Spacer length (distance from each ramet to the original plants) were measured according to their order (Dong et al., 1997; Li and Xie, 2009); the first order spacer referred to the rhizome between the original plants and the first ramets, and the second order spacer referred to the rhizome between the first and the second ramets. Biomass of different plant components (aboveground: shoots; belowground: rhizomes and roots) was measured after drying at 85°C for 48 h in an oven. Relative growth rate (RGR) was calculated using the following equations: RGR = (ln *w_2_* – ln *w_1_*) / (*t_2_* – *t_1_*), where *w_1_* was the initial biomass, *w_2_* was the biomass at harvest time, and (*t_2_* – *t_1_*) was the duration of the experiment.

### Data Analysis

The D’Agostino-Pearson omnibus test was used to analyze whether all factors were normally distributed. Data were square root or logarithm transformed, if necessary, to meet the assumptions of normality. Two-way ANOVAs, with the population size and burial depth as fixed factors, were performed to determine the main effects and interactions on biomass accumulation, relative growth rate, aboveground and belowground biomass allocation, length of the first and the second order spacers, number of new ramets, and number of clumping and spreading ramets. Multiple comparisons were applied by *post hoc* Tukey’s test at the 0.05 significance level. All analyses were performed using the software SPSS 17.0 for Windows (SPSS Inc., Chicago, IL, United States).

## Results

### Biomass Accumulation and Relative Growth Rate

**FIGURE 1:**
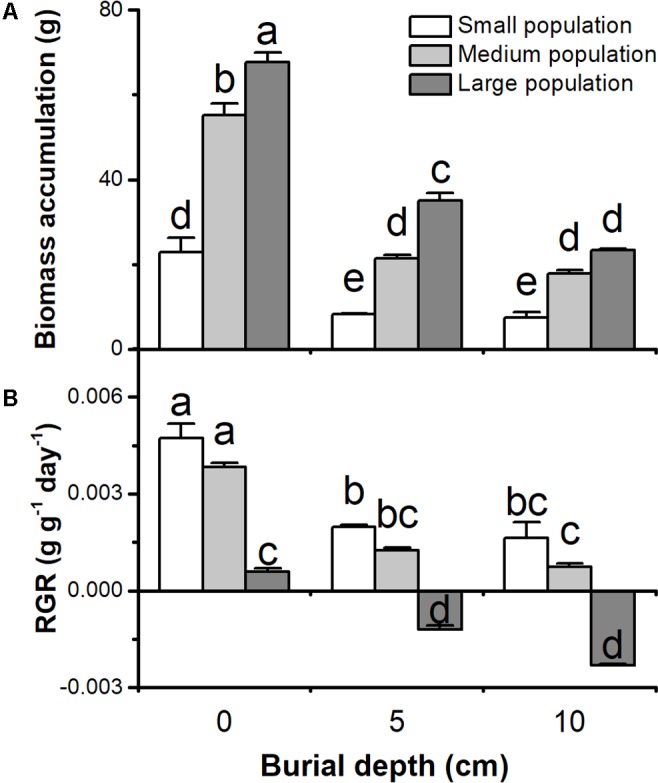
**(A)** Biomass accumulation and **(B)** relative growth rate (RGR) of three population sizes of *Carex brevicuspis* growing in three burial depths. Error bars indicate the standard error of the mean. Different letters indicate significant differences among different treatments (Tukey’s tests, α = 0.05).

Biomass accumulation and relative growth rate (RGR) of *C. brevicuspis* populations were significantly affected by both burial depth and population size, with significant interactions (*P* < 0.05, **Figure 1** and **Table 1**). Biomass accumulation and RGR were higher in the 0 cm burial treatments than in the 5 and 10 cm burial treatments (*P* < 0.05, **Figure 1**). Biomass accumulation in larger populations was higher than that in small or medium populations (*P* < 0.05, **Figure 1A**). However, RGR of the small and medium populations was especially high compared to the large populations (*P* < 0.05, **Figure 1B**). RGR was negative only in large populations under 5 or 10 cm burial conditions (-0.001 to -0.002 g g^-1^day^-1^, *P* < 0.05, **Figure 1B**). It was clear that sedimentation had negative effects on plant growth, and had larger negative effects on larger populations than smaller ones.

**Table 1:** Summary of two-way ANOVA analysis in three population sizes of *Carex brevicuspis* growing at three sedimentation depths.

Dependent variable	Burial	Population	B × P
	depth (B)	size (P)	
Biomass accumulation (g)	276.418***	200.808***	18.986***
Relative growth rate (g g^-1^ day^-1^)	140.901***	215.039***	1.319^ns^
Biomass allocation (%)	509.026***	29.996***	23.869***
Length of the first order spacer (cm)	73.108***	29.003***	16.762***
Length of the second order spacer (cm)	147.467***	40.798***	21.066***
Number of new ramets	25.708***	135.424***	4.776**
Proportion of ramets (%)	115.578***	12.788***	2.049^ns^
d. f.	2	2	4

*^∗∗∗^P < 0.001, ^∗∗^P < 0.01, ^∗^P < 0.05, ^*ns*^P > 0.05.*

### Biomass Allocation

**FIGURE 2:**
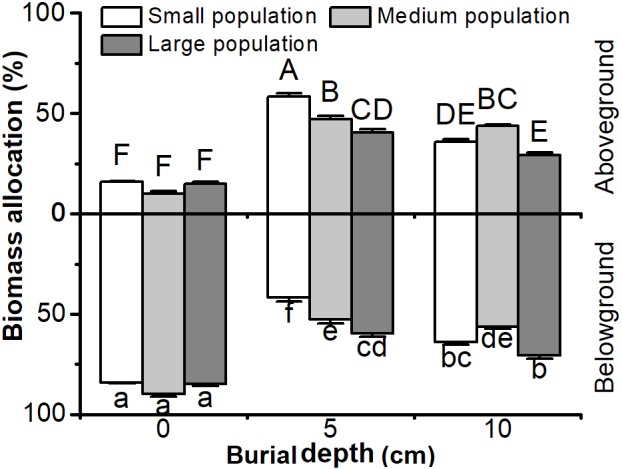
Biomass allocation to above- and belowground parts of *Carex brevicuspis*, growing in three population size at three burial depths. Error bars indicate the standard error of the mean. Different uppercase and lowercase letters indicate significant differences among different treatments (Tukey’s tests, α = 0.05).

Biomass allocation to aboveground or belowground parts in *C. brevicuspis* populations was significantly affected by both burial depth and population size, with significant interactions (*P* < 0.05, **Figure 2** and **Table 1**). Biomass allocation to belowground parts was higher than aboveground parts, except in small populations with 5 cm sediment treatments (*P* < 0.05, **Figure 2**). Under shallow or deep burial conditions, more biomass was allocated to belowground parts in larger populations than smaller ones (*P* < 0.05, **Figure 2**). Shallow or deep sediment burial led to more biomass being allocated to aboveground parts than in 0 cm treatments, and it was higher in 5 cm than 10 cm burial treatments (*P* < 0.05, **Figure 2**). It was clear that both smaller populations and shallower burial led to greater biomass allocation to aboveground parts.

### Spacer Length

**FIGURE 3:**
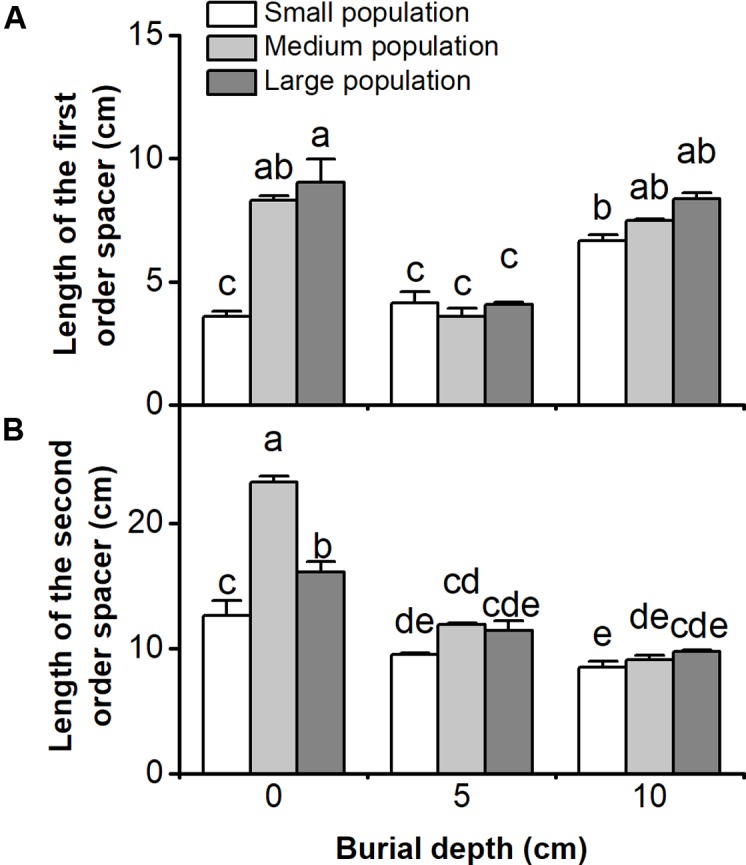
Lengths of **(A)** the first order spacer, and **(B)** the second order spacer of three population sizes and three burial depths of *Carex brevicuspis*. Error bars indicate the standard error of the mean. Different letters indicate significant differences among different treatments (Tukey’s tests, α = 0.05).

Length of the first and the second order spacers of *C. brevicuspis* populations were significantly affected by both burial depth and population size, with significant interactions (*P* < 0.05, **Figure 3** and **Table 1**). In 5 or 10 cm burial treatments, length of the first or the second order spacers did not differ according to population size (*P* > 0.05, **Figure 3**). The first order spacer was longer under deep burial than shallow burial, and it was longer in larger populations with 0 cm burial (*P* < 0.05, **Figure 3A**). Under shallow or deep burial conditions, length of the second order spacer was not significantly different and was lower than that in the 0 cm treatments; it was the longest in medium populations without sediment burial (*P* < 0.05, **Figure 3B**). It was clear that deep burial elongated the first order spacer more than shallow burial, and that sedimentation had negative effects on the growth of the second order spacers.

### Number of New Ramets

**FIGURE 4:**
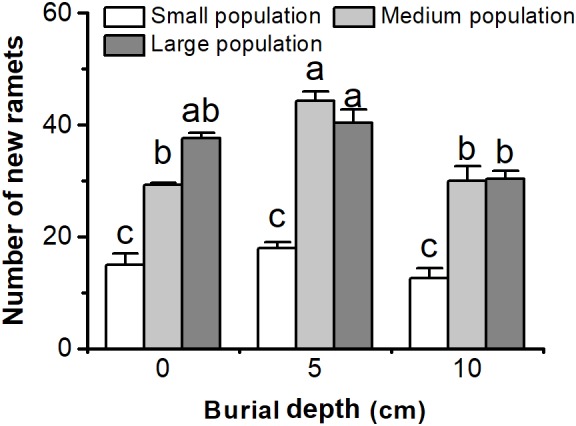
Number of new ramets of three population sizes of *Carex brevicuspis* growing in three burial depths. Error bars indicate the standard error of the mean. Different letters indicate significant differences among different treatments (Tukey’s tests, α = 0.05).

The number of new ramets of *C. brevicuspis* populations was significantly affected by both burial depth and population size (**Figure 4** and **Table 1**). The number of new ramets did not decrease with 5 or 10 cm burial depths, and larger populations usually had more new ramets than smaller ones (*P* < 0.05, **Figure 4**). The number of new ramets was the highest in the medium and large populations in the 5 cm burial treatments (*P* < 0.05, **Figure 4**). Sediment burial had no effect on the number of new ramets in small populations (*P* > 0.05, **Figure 4**).

### The Proportion of Clumping and Spreading Ramets

**FIGURE 5:**
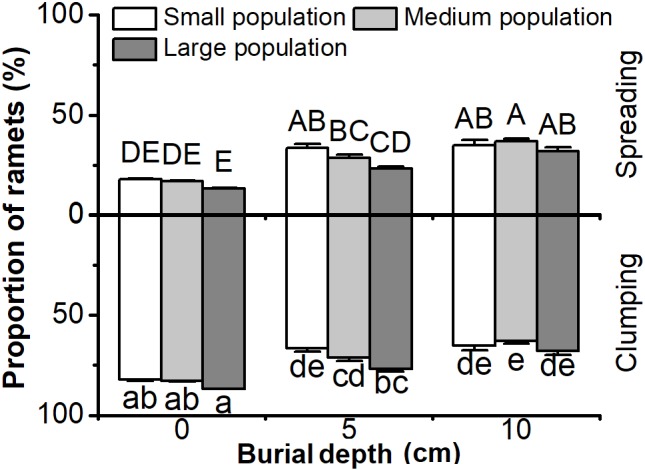
Proportion of clumping and spreading ramets of *Carex brevicuspis* for three population sizes and three burial depths. Error bars indicate the standard error of the mean. Different uppercase and lowercase letters indicate significant differences among different treatments (Tukey’s tests, α = 0.05).

The proportion of clumping or spreading ramets in *C. brevicuspis* populations was significantly affected by both burial depth and population size (*P* < 0.05, **Figure 5** and **Table 1**). The proportion of clumping ramets was higher than that of spreading ramets overall, while increasing burial depth from 5 to 10 cm led to an increase in the proportion of spreading ramets (*P* < 0.05, **Figure 5**). Larger populations had a higher proportion of clumping ramets than smaller populations in the 5 cm burial treatments (*P* < 0.05, **Figure 5**). It was clear that deeper burial and smaller populations led to an increase in the proportion of spreading ramets within *C. brevicuspis* populations.

## Discussion

In our treatments, space did not limit plant growth, since the plants did not reach the edge of the blocks before harvest. We evaluated the role of population size of *C. brevicuspis* in its response to sedimentation stress. Plant growth of *C. brevicuspis* populations were significantly affected by both sediment burial and population size, and the biomass accumulation and RGR of *C. brevicuspis* populations decreased under sediment burial conditions. Under deep burial conditions, our observations were consistent with those of previous studies (Pan et al., 2012; Li et al., 2016; Chen et al., 2017), which found that *C. brevicuspis* cannot acclimatize to heavy sedimentation. In this experiment, we did not find the stimulatory effect of shallow burial on plant growth, which has been reported previously by other studies (Pan et al., 2012; Chen et al., 2017). However, those studies were performed with single plants, and the stimulatory effect might be insignificant on larger population sizes. Additionally, the stimulatory effect might also be limited by intraspecific resource competition (Schwinning and Weiner, 1998; van Kleunen et al., 2001). Therefore, the responses to sedimentation might be different between single plants and ramet populations.

It is well known that larger populations may contain more physiologically integrated ramets, which can help them to endure sedimentation stress more than smaller populations or single ramets (Yu et al., 2001, 2004). However, the RGR of the small and medium populations were especially high compared to the large populations. Additionally, larger populations had more negative effects on growth than the smaller ones. These results indicated that smaller populations might help the species to withstand sedimentation stress, and are partly consistent with our hypothesis 1, which predicts that larger populations will have a higher biomass accumulation and RGR compared to other sizes of population. The negative effects of growing population size are consistent with previous studies (Ekstam, 1995; Horvitz and Schemske, 2002), in which RGR was negatively correlated with plant size. Other factors, such as plant density, light availability, and nutrient limitation (Schwinning and Weiner, 1998; van Kleunen et al., 2001; Li et al., 2014), may also limit plant growth. In fact, shelf-shading might cause a lower RGR of larger populations, similar to the previously reported lower RGR of larger plants (Poorter and Remkes, 1990). It seems that large population do not have a high integration capacity based on RGR.

Deep burial elongated the first order spacers more than shallow burial, which is consistent with previous research (Li and Xie, 2009; Chen et al., 2011). The elongation of rhizomes or other perennating organs, helps to escape burial stress, for photosynthesis and future growth (Maun, 1998; Shi et al., 2004; Yu et al., 2004). However, negative effects were found on the growth of the second order spacers under burial conditions. These effects might be caused by the larger cost to form longer spacers under deep burial conditions (Sammul, 2011). Additionally, length of the second order spacers in the 5 and 10 cm burial treatments was not different for different population sizes. The number of new ramets was not lower in the 5 or 10 cm burial treatments, and sediment burial had no effect on the number of new ramets in small populations. These results suggest that sufficient resource can be provided at the ramet population level to escape sedimentation stress (Maun, 1998; Deng et al., 2008; Luo and Zhao, 2015), even in small populations. Therefore, the second ramets might be free to grow at the new shallower depths reached by the first ramets.

Both shallow burial and smaller populations led to increased biomass allocation to aboveground parts. Under these conditions, plant growth might be limited by competition for soil nutrients (Li et al., 2014; Chen et al., 2017); therefore, the high ratio of biomass allocation to aboveground parts can enable plants growing on the sediment surface to acquire the most limiting resources, such as light and oxygen (Brown, 1997; Dech and Maun, 2006). However, biomass allocation to belowground parts was higher in deep burial treatments and large populations, indicating that competition for nutrient resources among clones is intense (Brown, 1997; Maun, 1998; Deng et al., 2008). Therefore, biomass allocation responses of clonal plants of different population sizes to sediment burial could be attributed to resource limitation.

In our experiment, deeper burial and smaller populations led to a higher proportion of spreading ramets of *C. brevicuspis*, which is partly consistent with our hypothesis 2. In response to heavy sedimentation, *C. brevicuspis* can change its growth form with a shift from clumping to spreading ramets (Ye et al., 2006; Chen et al., 2011). In contrast to a previous study (Chen et al., 2011), the proportion of clumping ramets was higher in all treatments at the ramet population level similar to natural conditions (Chen et al., 2014). These results were similar to the biomass allocation, with resource limitation of smaller populations inducing them to produce more spreading ramets to escape sedimentation, or to forage for resources (van Kleunen et al., 2001). The findings of the present study indicated that smaller populations of *C. brevicuspis* enabled the plants to adjust their escape responses to sedimentation stress. However, besides the depth of sediment burial and population size, growth of *C. brevicuspis* populations is influenced by other properties of sediments and populations. Further studies are required to clarify the interactive effects between sediment heterogeneity, population structure, and the intra- or interspecific competition on clonal growth of aquatic macrophytes.

## Author Contributions

B-HP and Y-HX wrote the manuscript text and executed the technical assays and statistical analysis. Y-HX designed the experiment and edited the manuscript text. B-HP, Y-HX, FL, Y-AZ, and Z-MD contributed to data collection and interpretation of the data. All authors reviewed the manuscript.

## Conflict of Interest Statement

The authors declare that the research was conducted in the absence of any commercial or financial relationships that could be construed as a potential conflict of interest.
